# Synthesis and Exploration of the Lubricating Behavior of Nanoparticulated Mo_15_S_19_ in Linseed Oil

**DOI:** 10.3390/ma11091783

**Published:** 2018-09-19

**Authors:** Ignacio A. Fernández-Coppel, Pablo Martín-Ramos, Jesús Martín-Gil, Ramón Pamies, Manuel Avella, María Dolores Avilés

**Affiliations:** 1 Engineering of Manufacturing Processes Group, School of Industrial Engineering, University of Valladolid, C/Francisco Mendizábal 1, 47014 Valladolid, Spain; ignacio.alonso.fernandez-coppel@uva.es; 2 Department of Agricultural and Environmental Sciences, EPS, Instituto de Investigación en Ciencias Ambientales de Aragón (IUCA), University of Zaragoza, Carretera de Cuarte, s/n, 22071 Huesca, Spain; 3 Agriculture and Forestry Engineering Department, ETSIIAA, Universidad de Valladolid, Avenida de Madrid 44, 34004 Palencia, Spain; mgil@iaf.uva.es; 4 Grupo de Ciencia de Materiales e Ingeniería Metalúrgica, Universidad Politécnica de Cartagena, Campus de la Muralla del Mar, 30202 Cartagena, Spain; ramon.pamies@upct.es (R.P.); mdolores.aviles@upct.es (M.D.A.); 5 Unidad de Microscopía Avanzada, Parque Científico UVa, Universidad de Valladolid, Paseo Belén 11, 47011 Valladolid, Spain; um.parque.cientifico@uva.es

**Keywords:** antifriction additives, linseed oil, lubricant, Mo_15_S_19_, nanoparticles

## Abstract

Molybdenum chalcogenides present interesting properties beyond their superconducting critical temperatures and upper critical magnetic fields, making them suitable for potential applications in tribology, batteries, catalysis, or thermopower. In this study, Mo_15_S_19_ nanoparticles with an average diameter of 10 nm were synthesized via the reaction of ammonium molybdate with hydrochloric acid and elemental sulfur as reducers at 245 °C. The oxidation to MoO_3_ in air was efficiently avoided by using linseed oil as a reaction medium and dispersant. Scanning electron microscopy (SEM) micrographs of the as-prepared samples revealed the presence of few-micron-size aggregates, while transmission electron microscopy (TEM) characterization evidenced that the samples were polynanocrystalline with a high degree of homogeneity in size (standard deviation of 2.7 nm). The absence of the first-order (00l) reflection in the X-ray diffraction pattern was also indicative of the absence of Mo_3_S_4_ stacking, suggesting that it was a non-layered material. A dispersion of the nanoparticles in linseed oil has been studied as a lubricant of steel–steel sliding contacts, showing the formation of a surface layer that reduces wear and mean friction coefficients with respect to the base oil.

## 1. Introduction

**Figure 1:**
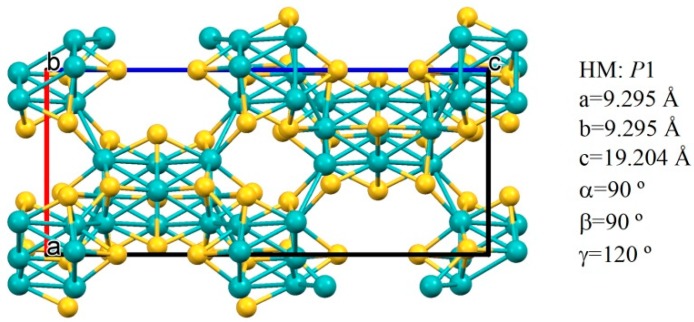
Mo_15_S_19_ structure, viewed along *b* axis. Plotted with Mercury [9] using the crystallographic information file (CIF) file available from the literature [3,4].

Mo_15_S_19_ is a polymorph of the famous Chevrel phases [1,2]. It is a metastable binary phase that has been described as dimeric, with full formula Mo_30_S_38_ [3,4] (Figure 1).

To the best of the authors’ knowledge, the direct synthesis of this phase has not been reported in the literature, including review papers [5]. The identification of Mo_15_S_19_ was reported by Tarascon and Hull [6] as a product of the oxidation of the In_3.3_Mo_15_S_19_ phase with HCl at low temperatures. The same authors preconized for Mo_15_S_19_ the possibility to undergo topotactic redox reactions with lithium or sodium or to react at low temperatures with low melting point ternary elements to form new ternary compounds [6], in a similar fashion to its Se homologues [7,8]. Thus, the open framework structure Mo_15_S_19_ can be deemed as an interesting system for intercalation studies [5].

Research on these compounds, discovered over 40 years ago, initially came from their superconducting critical temperature and upper critical magnetic field [10], which opened hopes for their use in the fabrication of magnets as a replacement for the well-known Nb–Sn superconducting magnets [11]. Other fundamental features were found, such as the coexistence of magnetic order with the superconducting state. Although these features are still of interest for the scientific community, other potential applications are now foreseen, such as their use in batteries (as cathode materials), in catalysis (for the hydrodesulfurization of thiophene and/or decomposition of nitrogen oxides), and in thermopower technology (due to their low thermal conductivity and high figures of merit ZT, i.e., their ability to efficiently produce thermoelectric power) [1].

The closest Chevrel phase to Mo_15_S_19_ is Mo_15_S_20_ (or Mo_3_S_4_). Mo_15_S_19_ gives Mo_15_S_20_ by thermal heating at temperatures above 500 °C [12]. Mo_3_S_4_ crystallizes in the hexagonal space group P6_3_/m and consists of an equal mixture of the original Mo_9_S_27_ cluster unit and the classical one Mo_6_S_8_S_6_ interconnected through Mo bonds [13].

The applications of molybdenum chalcogenides as lubricant additives and surface coatings to reduce friction coefficients and surface damage and wear of materials have been extensively studied [14,15,16,17]. Although emphasis has been placed on MoS_2_ polymorphs, interesting tribological properties have also been reported for other MoS*_x_*-based coatings, such as those based on aforementioned Mo_3_S_4_ [18]. These materials may pose an alternative to other nanostructure lubricants [19,20,21], in particular to those based on graphene [22,23,24,25,26,27,28].

In this paper, we report a route to synthetize Mo_15_S_19_ using linseed oil as a reaction medium and dispersant, with a view of obtaining a non-oxidized nano-particulated material. The friction and wear reducing ability of a dispersion of the nanoparticles in the environmentally-friendly vegetable linseed oil (one of the natural oils suggested to replace mineral oil in, for instance, drilling fluids [29]) has been assessed.

## 2. Materials and Methods

### 2.1. Materials

Ammonium molybdate tetrahydrate ((NH_4_)_6_Mo_7_O_24_·4H_2_O; CAS No. 12054-85-2; purity ≥99%) was purchased from Sigma-Aldrich Química S.L. (Madrid, Spain). Linseed oil was purchased as NaturGreen line oil from Laboratorios Almond S.L. (Murcia, Spain). Hydrochloric acid, sulfur, and hexane were commercially available analytical-grade products. All chemicals used in the experiment were used without further purification.

### 2.2. Synthesis

Mo_15_S_19_ nanoparticles were synthesized via the reaction of ammonium molybdate with hydrochloric acid and elemental sulfur as reducing agents, in linseed oil as the reaction medium. A total of 1.23 g of (NH_4_)_6_Mo_7_O_24_·4H_2_O (1 mmol) was stirred in 27 mL linseed oil, proceeding with the stirring of the mixture while 2 mL of hydrochloric acid fuming 37% was carefully added dropwise. Upon reaction, blue and green colors appeared. Subsequently, 0.256 g of sulfur powder S_8_ (1 mmol) was added and a change in color to red occurred. The system was heated under stirring at 245 °C for 100 min until a black color appeared. Aliquots were taken from the reaction flask and were allowed to cool down to room temperature, and the resulting nano-Mo_15_S_19_ was then collected by centrifugation by adding excess hexane–methanol–water (1:1:1) three times. The as-prepared Mo_15_S_19_ (slightly soaked in linseed oil) was a stable product and the corrosive effects of chloride remained minimized by the use of hexane–hydromethanol as the washing liquid.

For tribological tests, and to avoid the potential corrosive effects of halides on metals, an entirely chloride-free lubricant may be obtained by sonication of 100 mL of a 0.0195 M solution of Mo_15_S_19_ nanoparticles in linseed oil (prepared according the previous procedure) for five periods of 5 min each at 60 °C, using 20 mL of a methanol/water mixture (1:1, v/v) each time.

A probe-type UIP1000hdT ultrasonicator (Hielscher, Teltow, Germany; 1000 W, 20 kHz) was used for the sonication of solutions.

### 2.3. Characterization

The as-prepared Mo_15_S_19_ was characterized by Fourier-transform infrared spectroscopy (FTIR), scanning electron microscopy (SEM), transmission electron microscopy (TEM), and X-ray powder diffraction (XRPD).

The vibrational spectrum in the 400–4000 cm^−1^ spectral range was characterized using a Thermo Scientific (Waltham, MA, USA) Nicolet iS50 FT-IR Spectrometer, equipped with an in-built diamond attenuated total reflection (ATR) system, with a 1 cm^−1^ spectral resolution and 64 scans.

X-ray powder diffraction patterns were obtained using a Bruker (Billerica, MA, USA) Discover D8 powder diffractometer in a Bragg–Brentano geometry.

Scanning electron microscopy (SEM) and transmission electron microscopy (TEM) images were collected with an FEI (Hillsboro, OR, USA) Quanta 200FEG microscope and with a JEOL (Akishima, Tokyo, Japan) JEM-FS2200 HRP microscope, respectively.

### 2.4. Rheological and Tribological Study

Viscosity measurements of the base linseed oil and of the dispersion of nanoparticles in linseed oil were performed with an AR-G2 rotational rheometer (TA instruments; New Castle, DE, USA), using a plate-on-plate configuration. The shear flow influence on the viscosity was determined at 25 °C by changing the shear rate from 10^−2^ to 500 s^−1^. The influence of temperature on viscosity was studied from 25 °C to 100 °C under a constant shear rate of 50 s^−1^.

Tribological tests according to ASTM G-99 standard were carried out in a pin-on-disk ISC 200PC tribometer (Implant Science Co; Wakefield, MA, USA) under ambient conditions (22 ± 1 °C; RH: 50–55%). The materials used in the tribological tests were stainless steel balls (nominal composition in weight percentage: <0.03 C; 16−18.5 Cr; 10−14 Ni; 2−3 Mo; <2 Mn; <1 Si; <0.045 P; <0.03 S; balance Fe; hardness: 195 HV) with 1.5 mm sphere radius and AISI 316L stainless steel disks (25 mm diameter; 2.5 mm thickness; surface roughness Ra 0.12−0.13 μm). The disk surface was completely covered with the lubricant before applying a normal load of 0.49 N, at a speed of 0.1 m·s^−1^ (sliding radius 9 mm), for a sliding distance of 372 m (sliding time: 1 h). The materials were washed with *n*-hexane and dried in air, before and after each test. Wear tracks sections and surface topography were determined with a Talysurf CLI 3D optical profiler (AMETEK; Berwyn, PA, USA). Friction coefficients in each test were calculated as average values from the entire duration of the test. Final friction coefficients and wear rates were calculated as mean values after at least three tests under the same conditions. The cross sectional profiles of the wear tracks were obtained from an average of 300 profiles, each of which consisted of 300 points.

## 3. Results and Discussion

### 3.1. Vibrational Characterization

**Figure 2:**
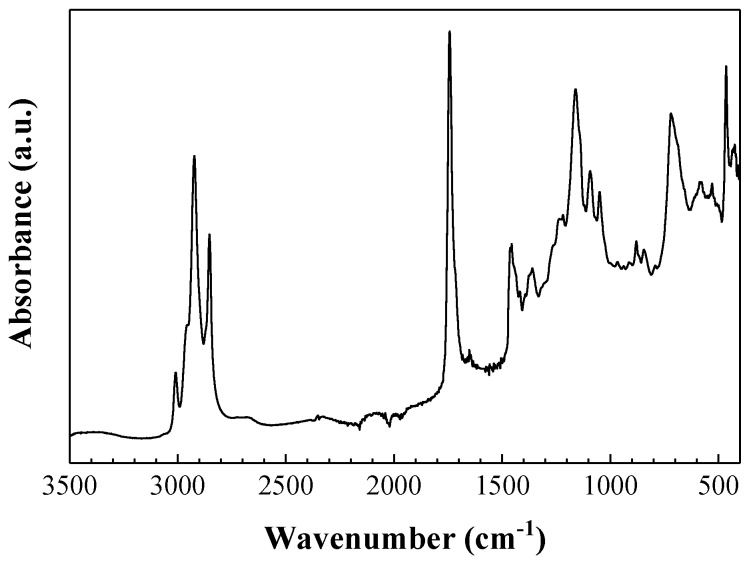
Attenuated total reflection (ATR)-Fourier-transform infrared spectroscopy (FTIR) spectra of Mo_15_S_19_ nanoparticles dispersion in linseed oil.

ATR-FTIR spectra were acquired to investigate the Mo_15_S_19_–linseed oil interaction. In the spectra depicted in Figure 2, absorption bands characteristic of linseed oil [30] appeared at 2923 cm^−1^ (H-C-H asymmetric) and 2853 cm^−1^ (H-C-H symmetric); 3010 cm^−1^ (C=C-H asymmetric stretching); 1742 cm^−1^ (C=O stretching); 1459–1456 cm^−1^ (asymmetric CH_3_ bending); 1240, 1164, and 1095 cm^−1^ (characteristic triglyceride ester triplet); and 720 cm^−1^ ((CH_2_)_n_ rocking/cis-(C-H)=CH wagging).

Mo_15_S_19_ characteristic bands appear at 579–586 cm^−1^ (*ν*(S-S) stretching mode, suggesting some double-bond character); 528 cm^−1^ (also attributed to *ν*(S-S) stretching modes); and 464 cm^−1^ (assigned to *ν*(Mo-S) stretching) [31].

No appreciable interactions were evidenced between linseed oil and Mo_15_S_19_ from the vibrational spectra, although some shifts could be observed for three (weak) linseed oil bands: 16 cm^−1^ for the *ω*(CH_2_) band at 1376 cm^−1^ (vs. 1360 cm^−1^ in the spectrum of the mixture), 19 cm^−1^ for the band at 1237 cm^−1^ associated with *ν*(C-O) in triglycerides ester linkage (vs. 1218 cm^−1^ in the spectrum of the mixture), and 19 cm^−1^ for the band at 1068 cm^−1^ related to *ν*(C-O-C) ethers (vs. 1049 cm^−1^ for the mixture) [32].

### 3.2. X-ray Powder Diffraction Analysis

**Figure 3:**
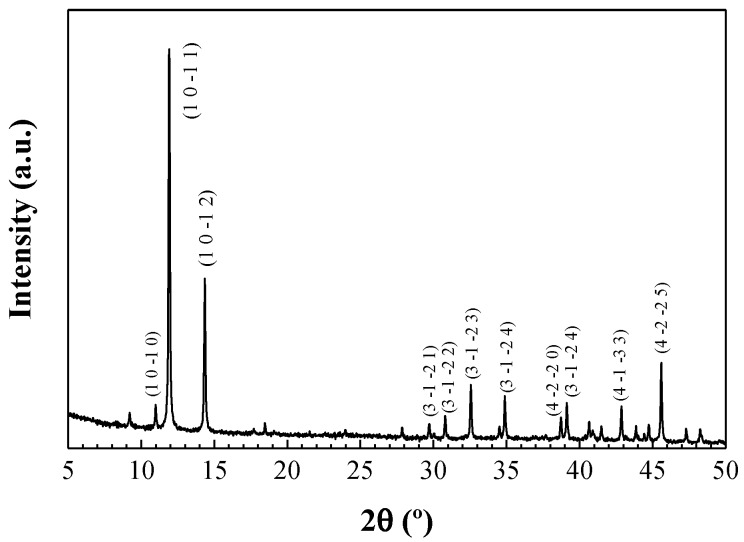
Experimental X-ray powder diffraction patterns for Mo_15_S_19_ nanoparticles. Four-index notation of the Miller indices has been used to allow direct comparison with the diffractograms reported in the literature [3,4].

The intensity peaks at 11.04, 12.5, 14.3, 43.04, and 45.4° can be ascribed to hexagonal Mo_15_S_19_ (JCPDS card # 40-0936) in good agreement with the calculated pattern (Figure 3).

### 3.3. SEM Analysis

**Figure 4:**
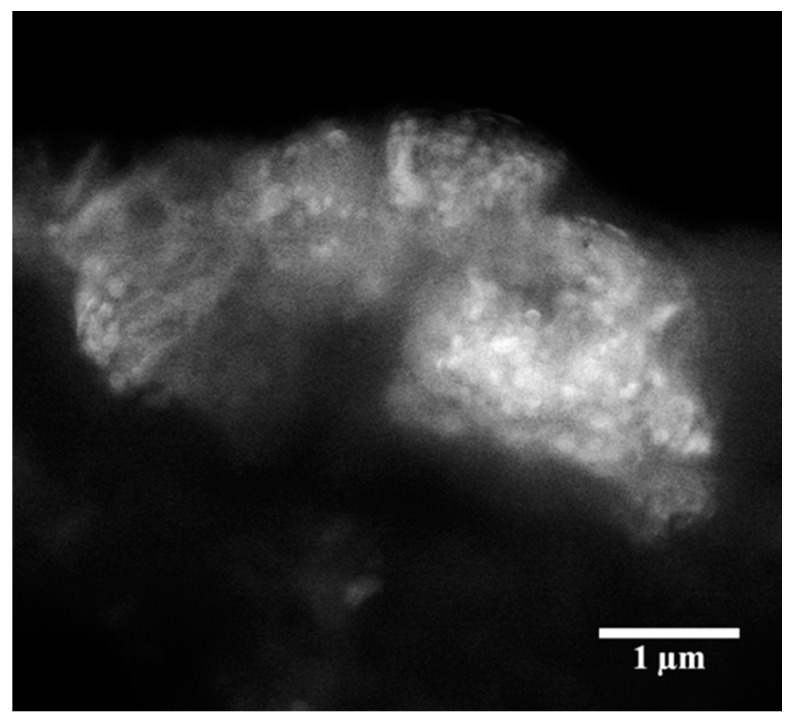
Scanning electron microscopy (SEM) micrograph of as-prepared Mo_15_S_19_ particles showing their aggregation state prior to sonication.

The morphology analysis of the synthesized Mo_15_S_19_ revealed the presence of few-micron-size aggregates, such as the one depicted in Figure 4. These would result from the tendency of unmodified nanoparticles to settle down after a finite time if the oil is kept stationary, a behavior that can be minimized by using surfactant-modified nanoparticles [33].

As the magnification in the SEM was insufficient to clearly show the nano-particulate nature of the material, samples were sonicated and subjected to TEM analysis. The sonication of oil dispersions has been reported to significantly improve the dispersion quality compared with mechanical shaking and stirring, although it would not completely disaggregate nanoparticles, according to Mosleh, et al. [34].

### 3.4. SAED Pattern and TEM Images

**Figure 5:**
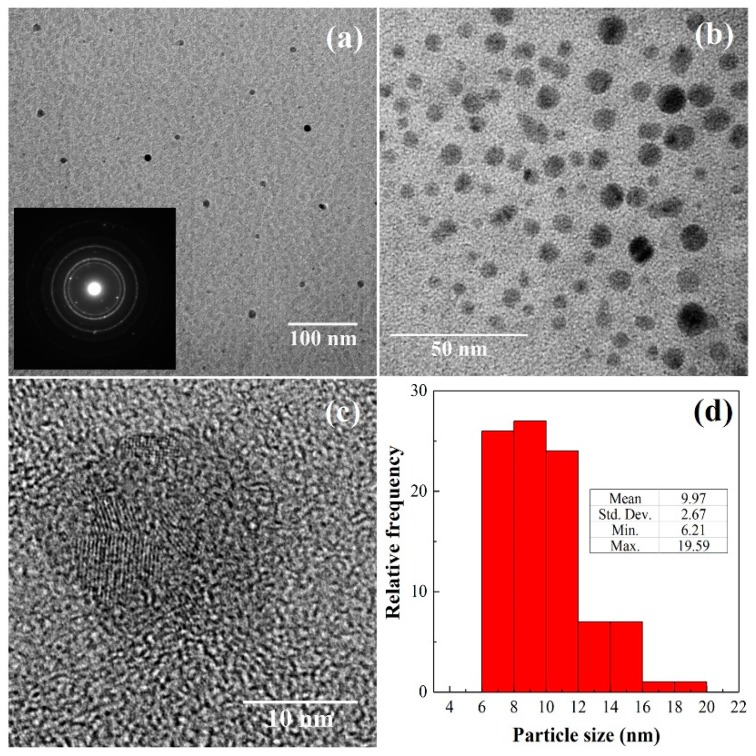
(**a**–**c**) Transmission electron microscopy (TEM) micrographs of Mo_15_S_19_ prepared in linseed oil at different magnifications. The inset in (**a**) shows the selected area electron diffraction (SAED) pattern. (**d**) Histogram showing the particle size distribution.

The selected area electron diffraction (SAED) pattern (inset in Figure 5a), compatible either with nanoparticles of single-crystalline structure or with a poly-nanocrystalline sample, was not conclusive. Close inspection of TEM images of as-prepared Mo_15_S_19_ particles dispersed in methanol (Figure 5a*–*c) evidenced their nanoscale size, with an average diameter of 10 nm (standard deviation of 2.7 nm, Figure 5d), smaller than the 70 nm diameter reported in Rosentsveig, et al. [35]; than the 50 nm diameter reported in Santillo, et al. [36]; and than the 10–30 nm diameters found by Duphil, et al. [37] for MoS_2_. Only in the work by Yu, et al. [38], smaller diameters (5 nm) were attained. However, it is worth noting that, according to Kogovšek and Kalin [39], the NPs size and morphology would not significantly affect the coefficient of friction, which would mostly depend on the NPs’ material.

### 3.5. Rheological Study

**Figure 6:**
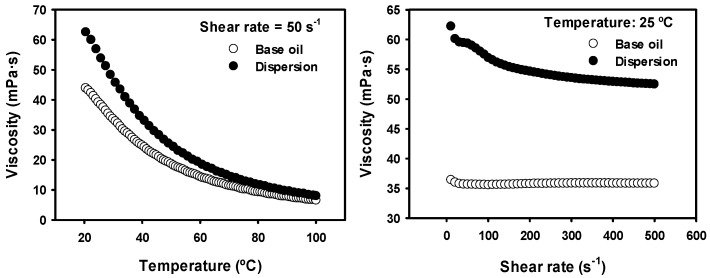
Viscosity variation with temperature and with shear rate for both lubricants.

The variation of the viscosity of the base linseed oil and of the dispersion of Mo_15_S_19_ in the base oil with increasing temperature under constant shear rate (50 s^−1^) and with increasing shear rate under constant temperature (25 °C) are shown in Figure 6. In both samples, there was an expected decrease of the viscosity with the increasing values of temperature. The dispersion showed higher viscosity values than those of the base oil, because of the presence of particles in the fluid, which would act as obstacles, and thus the fluid presented a higher resistance to flow. Only at 100 °C, the viscosity values of both fluids became similar. While the base oil showed a Newtonian behavior, the dispersion showed a slight decrease of its viscosity over the whole range of shear rate investigated. This shear thinning effect can be attributed to the disruption of agglomerates [40].

### 3.6. Tribological Study

As shown in Table 1, the dispersion was able to reduce the friction coefficient by 23.4% with respect to the base oil. In each test, the friction coefficient was calculated as an average of all the friction values recorded from the entire test duration.

**Figure 7:**
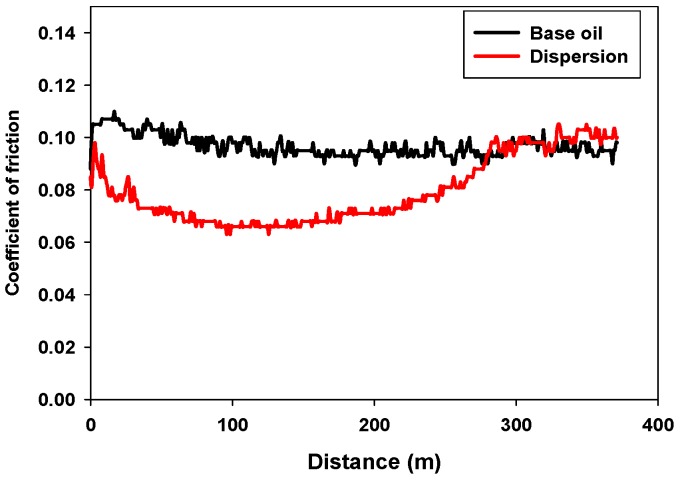
Variation of coefficient of friction (COF) with sliding distance for the base oil (*black*) and the dispersion (*red*).

Figure 7 shows the evolution of the coefficient of friction (*µ*) with sliding distance for both lubricants. While the neat base oil (Figure 7, black) showed a constant friction value, the dispersion was able to reduce friction values during 300 m, to reach a final friction value similar to that of the base oil (Figure 7, red). This result could be related with the stability of the dispersion and with the increasing values of viscosity when nanoparticles are added, as shown in Figure 6. The formation of agglomerates and the deposition of the nanoparticles on the sliding surfaces could account for the final friction increase.

Regarding the behavior of the base oil, from an analysis of the scoring resistance of oils according to the welding load and load wear index, Grigoriev, et al. [41] showed that, according to GOST9490-75, linseed oil featured better tribotechnical properties than those of I20А industrial basic oil. They concluded that it was most likely that the observed advantage of linseed oil was related to the high content of linolenic acid triglycerides, which is intrinsic for a relatively large number of unsaturated bonds, in contrast with, for instance, coconut oil (assayed as a nanolubricant with MoS_2_ nanoparticles by Koshy, Rajendrakumar and Thottackkad [33]).

The *µ* value for the dispersion was similar to that obtained for acrylated epoxidized linseed oil (AELO) based bio-nanocomposites with low TiO_2_ content (1 wt.%) [42]. It is also worth noting that the final value was comparable to the best value reported for linseed oil bonded to mercaptosilane treated aluminum [43]. Nonetheless, it should be noted that because the tribological tests in these two studies were performed under different operating parameters as in the present study, comparisons of the coefficients of friction above should be taken with caution.

The tribological performance of the Mo_15_S_19_–linseed oil dispersion may be explained by taking an alternative view of it as a nanocomposite fluid based on the vegetable oil with Mo_15_S_19_ as a nano-reinforcement, in a similar way to the aforementioned linseed oil–TiO_2_ green nanocomposites reported by Díez-Pascual and Díez-Vicente [42], for which strong reductions in the coefficient of friction and the wear rate were attained with the highest TiO_2_ content. In both cases, the dispersed inorganic particles would reduce the free volume and the mobility of the oil polymer chains, leading to more compact networks. As in the case of linseed oil–TiO_2_, the presumably high thermal conductivity of Mo_15_S_19_ would enable a rapid heat dissipation, hence resulting in a lower temperature in the sliding contact.

### 3.7. Surface Analysis and Wear Mechanisms

**Figure 8:**
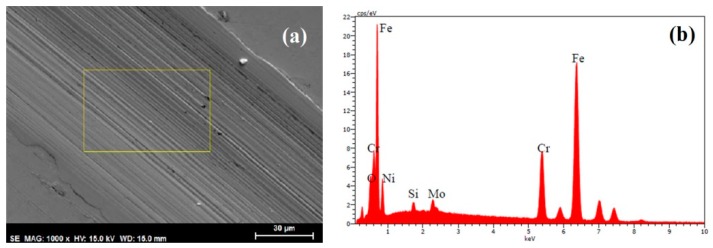
(**a**) SEM micrograph of the wear track on AISI 316L stainless steel disk after lubrication with base oil at 1000× magnification and (**b**) EDX spectrum of the selected area.

Figure 8a shows an SEM micrograph of the wear track on the surface of the AISI 316L stainless steel disk after lubrication with the base oil, while Figure 8b depicts the results of the energy dispersive x-ray spectrometry (EDX) analysis of the selected area, showing the composition of the stainless steel. The severe wear was the result of plastic deformation, as may be observed on the edges of the wear track, and the result of abrasion, as evidenced by the parallel abrasion grooves along the wear track, parallel to the sliding direction.

**Figure 9:**
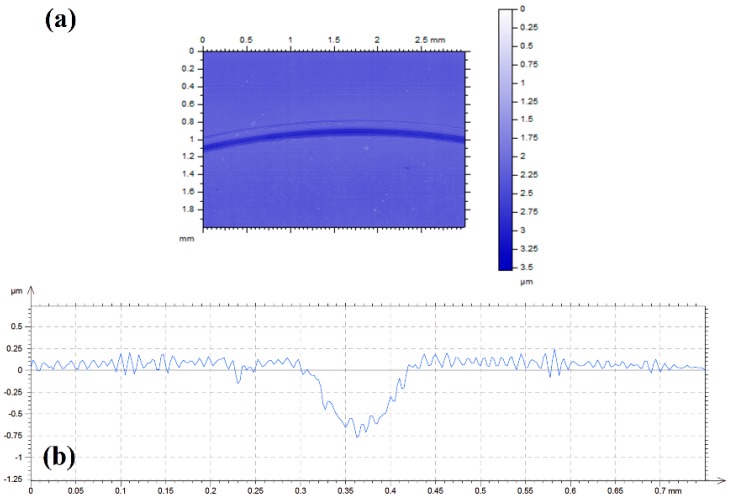
(**a**) Surface topography and (**b**) cross sectional profile of the wear track on AISI 316L disk after lubrication with base oil.

The severity of the surface damage was shown by the surface topography profilometry image of a 3 mm × 2 mm section of the wear track, in which the origin was set on the highest value (Figure 9a). The cross sectional profile of the wear track is shown in Figure 9b. The average total wear volume after three tests was 1.23 × 10^−3^ mm^3^ (standard deviation 7.7 × 10^−4^).

**Figure 10:**
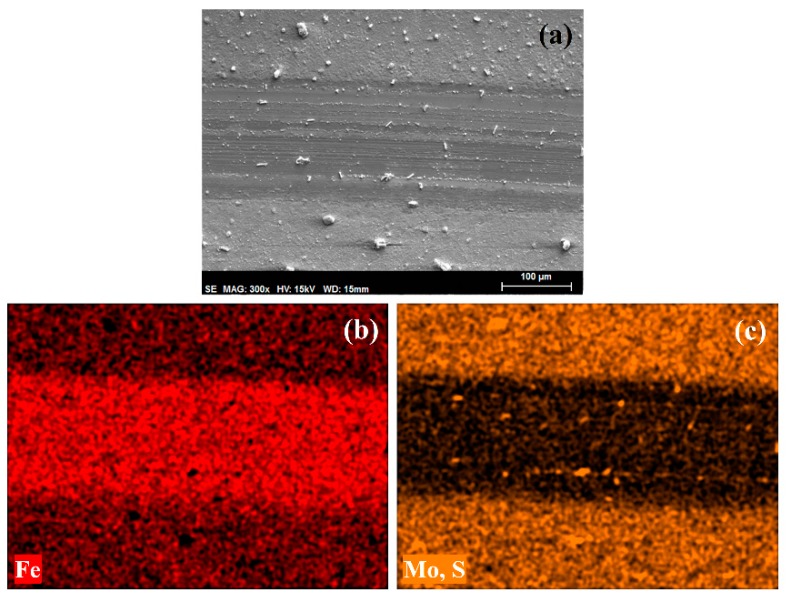
(**a**) SEM micrograph and (**b**,**c**) element maps of the wear track on AISI 316L disk after lubrication with the dispersion of Mo_15_S_19_ nanoparticles in linseed oil.

In Figure 10a, the SEM micrograph of the wear track on the steel surface after lubrication with the dispersion is shown. Analogously to the wear track seen after lubrication with the base oil in absence of nanoparticles, the presence of edges and parallel abrasion grooves along the track evidenced a similar, but significantly less pronounced, plastic deformation due to abrasion. Interestingly, the iron and molybdenum and/or sulphur element maps (Figure 10b) showed that a Mo and S rich layer covered the surface outside the wear track, with some particles inside the wear path. The presence of nanoparticles on the wear path may point to two mechanisms of reducing asperity contact: by filling the valleys of contacting surfaces and by the easy shearing of trapped solid lubricant nanoparticles at the interface without the formation of an adhered film [34,44].

**Figure 11:**
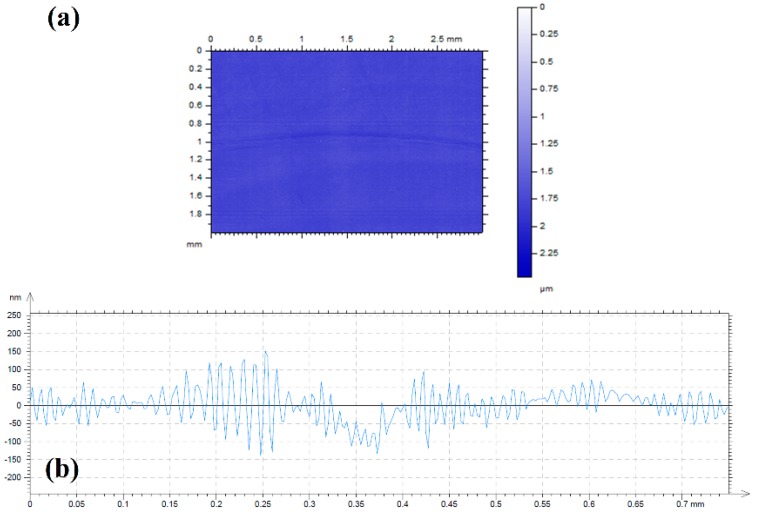
(**a**) Surface topography and (**b**) cross sectional profile of the wear track on AISI 316L disk after lubrication with the dispersion of nanoparticles in linseed oil.

The surface topography (Figure 11a) showed very mild surface damage. In fact, the cross sectional profile (Figure 11b) did not show any material loss, as the surface profile was very similar inside and outside the wear path (notice the nanometer scale). The removed wear volume could not be reliably determined, making it impossible to compare the improvement in the normalized wear coefficients with those reported in the studies by Tomala, et al. [45] and Paskvale, et al. [46] on MoS_2_ nanotubes in other oils.

**Figure 12:**
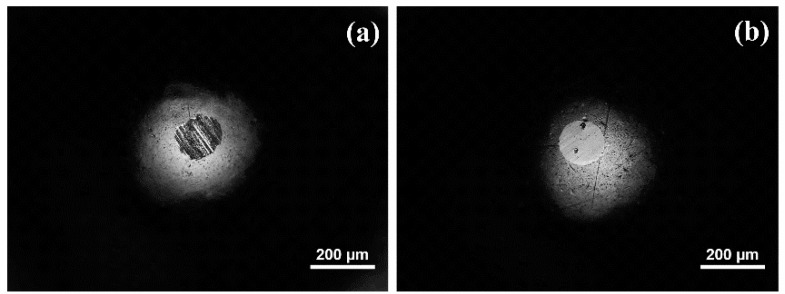
Wear scars on AISI 316L balls: (**a**) lubricated with base oil; (**b**) lubricated with the dispersion.

Stainless steel balls also showed wear scars at the end of the tests (Figure 12). In agreement with the different wear mechanisms described for steel disks, the wear scar on the ball after lubrication with base oil showed abrasive wear (Figure 12a), while a smooth circular scar—free from adhered material—was observed after lubrication with the dispersion (Figure 12b).

## 4. Conclusions

A soft chemical route to synthetize Mo_15_S_19_ using linseed oil as the reaction medium was presented, which yielded a non-oxidized nano-particulated material. The resulting Mo_15_S_19_ nanoparticles featured a regular size (with an average diameter of 10 nm) and some aggregation degree, while oxidation to MoO_3_ in air was efficiently avoided by linseed oil dispersant. With a view to assess their applicability as a lubricant additive and/or surface coating, rheological and tribological studies were conducted for a dispersion of the nanoparticles in linseed oil. The dispersion showed higher viscosity values than those of the base oil, and was found to reduce the friction coefficient by 23% with respect to the base oil. While the base oil displayed a Newtonian behavior, the dispersion presented a mild shear thinning effect probably because of the fraction of aggregates observed in SEM. The friction reduction lasted for only 300 m, and afterwards, the coefficient of friction increased to the value of the base oil. Tribological test results showed that the dispersion formed a surface layer that protected stainless steel surface against wear. The higher viscosity of the dispersion with respect to the base oil could account for its higher ability to support the applied load and to separate more effectively the sliding surfaces. This factor, together with the deposition of a layer of particles from the dispersion on the steel surface, would be responsible for the noticeable wear reduction observed. Further research to optimize the Mo_15_S_19_ loading, and to prevent the formation of agglomerates and the deposition of the nanoparticles on the sliding surfaces, is underway.

## Figures and Tables

**Table 1:** Average values and standard deviations (in brackets) of the coefficient of friction across three tests.

**Lubricant: Base Oil**	**Coefficient of Friction**
Test 1	0.097 (±0.004)
Test 2	0.115 (±0.007)
Test 3	0.109 (±0.003)
**Average**	**0.107 (±0.009)**
**Lubricant: Dispersion**	**Coefficient of Friction**
Test 1	0.081 (±0.015)
Test 2	0.079 (±0.013)
Test 3	0.085 (±0.010)
**Average**	**0.082 (±0.003)**
